# Evaluation of Weight-Based Co-trimoxazole Dosing in a Saudi Tertiary Hospital

**DOI:** 10.7759/cureus.47400

**Published:** 2023-10-20

**Authors:** Samah Alshehri, Rawan Alghuraybi, Elaf Ayoub, Jomana Bokhary, Manar Lashkar, Mohannad Alshibani, Khalid Eljaaly

**Affiliations:** 1 Clinical Pharmacy, King Abdulaziz University, Jeddah, SAU

**Keywords:** weight-based dosing, pjp, pneumocystis jiroveci pneumonia, dose, stenotrophomonas maltophilia, pneumocystis jirovecii, trimethoprim, co-trimoxazole

## Abstract

Background: Infections caused by *Stenotrophomonas maltophilia* (*S. maltophilia*)* *and *Pneumocystis jirovecii* (*Pneumocystis jirovecii* pneumonia (PJP)) require weight-based dosing for co-trimoxazole. The aim of this study is to assess the appropriateness of co-trimoxazole dosing in adult inpatients for the treatment of these infections.

Methodology: This is a single-center, cross-sectional study that included adult inpatients treated with co-trimoxazole for a weight-based dose indication (*S. maltophilia* and PJP). The primary outcome was the appropriateness of co-trimoxazole dosing for these infections.

Results: Forty-three patients were included in the study. Of the 43 patients, 29 (67.4%) were using co-trimoxazole for PJP treatment, and 14 (32.6%) were using it for *S. maltophilia* treatment. The co-trimoxazole dose was appropriate in 22 (51.2%) patients, 21 (72.4%) in the PJP treatment group, and one (7.1%) in the *S. maltophilia* treatment group. Underdosing was observed in 21 (48.8%) patients, of whom eight (27.6%) were in the PJP treatment group and 13 (92.9%) were in the *S. maltophilia* treatment group.

Conclusions: This study found a relatively high rate of underdosing of co-trimoxazole based on weight in hospitalized adults with PJP and *S. maltophilia* infections.

## Introduction

Co-trimoxazole is a combination of two antibiotics, trimethoprim (TMP) and sulfamethoxazole (SMX), and has a central role in the treatment of a variety of infections [1-3]. This synthetic combination of antibiotics shows a strong synergistic effect in which the sulfamethoxazole works by inhibiting the production of dihydropteroate from the two folate precursors, p-aminobenzoic acid (PABA) and 6-hydroxymethyl-7,8-dihydropterin pyrophosphate (DHPPP), while trimethoprim inhibits dihydrofolate (DHF) reduction into tetrahydrofolate (THF) [4]. Co-trimoxazole is active against some gram-negative organisms, such as enterobacterales, and gram-positive organisms, such as staphylococci and streptococci. It is used to treat urinary tract infections and skin and soft tissue infections. It is also used for the treatment of opportunistic infections, including those caused by *Stenotrophomonas maltophilia* (*S. maltophilia*), *Toxoplasma gondii*, and *Pneumocystis jirovecii* [5, 6]. *Stenotrophomonas maltophilia* is a gram-negative bacteria that was previously known as *Pseudomonas maltophilia* or *Xanthomonas maltophilia*. This bacteria most commonly causes respiratory infections; it also causes bloodstream infections, urinary tract infections, endocarditis, meningitis, and intra-abdominal infections [7]. The main therapeutic option for *S. maltophilia* is co-trimoxazole. The typical recommended dose is a relatively high dose administered as 12-15 mg/kg/day of trimethoprim moiety in divided doses either orally (PO) or intravenously (IV) [8].

*Pneumocystis jirovecii* pneumonia (PJP) is a life-threatening pulmonary infection. Patients who are at a high risk of contracting PJP include those with human immunodeficiency virus (HIV) with a low CD4 count, hematologic malignancies, organ transplantation, and patients receiving immunosuppressant medications [9, 10]. Co-trimoxazole is considered the most active option for PJP. This regimen is dosed as high as 15-20 mg/kg/day of trimethoprim in three to four daily divided doses, either per oral (PO) or intravenous (IV) [10, 11].

The combined antimicrobial agents are administered in a ratio of 1:5 (TMP to SMX). Oral formulations are available as single (80/400mg) and double (160/800mg) strength tablets. There is a discrepancy when a co-trimoxazole dose is ordered; for example, in the United States, when the ordered dose is 960 mg two times per day, this would refer to 960/4800 mg, which is equal to six double-strength tablets given two times a day. In other countries, this dose would refer to 160/800 mg, which means only one double-strength tablet given two times a day. [5, 12]. This discrepancy may lead to a dosing error since the dose did not elucidate whether it referred to the trimethoprim moiety or to the combined antimicrobial products. Therefore, standardization of the co-trimoxazole dose is essential to ensure the right dose is administered to the patient in order to achieve the desired antimicrobial effect [5, 12]. To avoid this error, doses should be standardized and clarified if they refer to the trimethoprim moiety or to the combined product [12]. The aim of this study is to assess the appropriateness of co-trimoxazole dosing in inpatient settings for the treatment of indications that require weight-based dosing in adults (PJP and *S. maltophilia* infections).

## Materials and methods

This was a cross-sectional study conducted at King Abdulaziz University Hospital (KAUH) in Jeddah, Saudi Arabia. The study was conducted from January 2017 to December 2018 and was approved by the Unit of Biomedical Ethics Research Committee at King Abdulaziz University, Jeddah, Saudi Arabia (approval number: 10-20). The inclusion criteria for the study were patients who were 18 years of age or older and received co-trimoxazole as an inpatient medication during their hospital stay. The indication for co-trimoxazole treatment had to be a weight-based dose, specifically for *S. maltophilia *infections and PJP. Patients who were under 18 years of age, those who received co-trimoxazole for indications other than *S. maltophilia* and PJP treatment, and patients with insufficient data in their hospital records were excluded from the study.

To collect the necessary data, the researchers extracted information from the electronic hospital records for each patient. The data included the patient's age, gender, actual weight, and creatinine clearance at the time the medication was ordered. Additionally, details about the co-trimoxazole medication were collected, such as its strength, dose, date of the initial dose, frequency, route of administration, and the indication for using the medication in these patients. To ensure accurate data entry, Google Sheets (Google LLC, Mountainview, CA) and Microsoft Excel version 16.43 (Microsoft Corp., Redmond, WA) were utilized. The primary outcome of the study was to assess the appropriateness of co-trimoxazole dosing for *S. maltophilia* infections and PJP. The assessment of dosing appropriateness was based on the indication for treatment and the patient's kidney function during their hospital admission.

For patients with PJP, a dose range of 15-20 mg/kg/day of trimethoprim was considered appropriate if the patient had normal kidney function [10, 11]. In the case of *S. maltophilia *infection, a dose range of 12-15 mg/kg/day of trimethoprim was deemed appropriate for patients with normal kidney function [8]. The doses were rounded to match the available dosage forms (single- and double-strength tablets for the PO doses and vial size for the IV doses). Patients requiring renal dose adjustments were also taken into account during the analysis. Statistical analysis was performed using the IBM Statistical Package for the Social Sciences software, version 24 (IBM Corp., Armonk, NY). The categorical data were presented as frequency and percentage, while continuous data were presented as mean +/-SD. The results of the analysis would provide insights into the appropriateness of co-trimoxazole dosing for *S. maltophilia* infections and PJP in relation to the indication and kidney function of the patients.

## Results

The screening started with 455 electronic medical records and was downsized to a sample size of 43 patients on weight-based co-trimoxazole doses. Of the 43 patients, 29 (67.4%) were using co-trimoxazole for PJP treatment, and 14 (32.6%) were using it for *S. maltophilia* treatment. The patients’ characteristics are summarized in Table 1.

**Table 1:** Patient characteristics SD: standard deviation; N: number; CrCl: creatinine clearance; PJP: *Pneumocystis jirovecii *pneumonia; *S. maltophilia*: *Stenotrophomonas maltophilia*

Characteristics (N=43)	Values
Baseline characteristics	
Age, years (mean ± SD)	49.1 ± 15.2
Weight, Kg, (mean ± SD)	63.1 ± 18.8
Gender, n (%)	
Male	23(53.5%)
Female	20(46.5%)
CrCl in mL/min, n (%)	
30 mL/min or above	37(86.0%)
Below 30 mL/min	6(14.0%)
Route of administration, n (%)	
Intravenous route	23 (53.5%)
Oral route	20 (46.5%)
Indication, n (%)	
PJP treatment	29 (67.4%)
*S. maltophilia* treatment	14(32.6%)

The mean +/-SD age of the study subjects was 49.1 ± 15.2 years, while the mean +/-SD weight was 63.1 ± 18.8 kg. There were 23 males included in the study, who accounted for 53.5% of the study population. With regards to kidney function in all patients, six (14.0%) patients had creatinine clearance below 30 mL/min and required co-trimoxazole dose adjustment. Co-trimoxazole was administered via the intravenous route in 23 (53.5%) patients, while 20 (46.5%) patients received it via the oral route.

The co-trimoxazole dose was appropriate in 22 (51.2%) patients, 21 (72.4%) in the PJP treatment group, and one (7.1%) in the *S. maltophilia* treatment group. Underdosing was observed in 21 (48.8%) patients, of whom eight (27.6%) were in the PJP treatment group and 13 (92.9%) were in the *S. maltophilia* treatment group. Out of eight underdosed PJP patients, six received co-trimoxazole intravenously, and two received it orally. Of the *S. maltophilia* patients, eight received it intravenously and five received it orally. No overdosing was observed in any group (Table 2).

**Table 2:** Co-trimoxazole dosing appropriateness N: number; PJP: *Pneumocystis jirovecii* pneumonia, *S. maltophilia*: *Stenotrophomonas maltophilia*

Dosing	n (%)
Overall dosing (N=43)	
Appropriate dose	22 (51.2%)
Underdose	21 (48.8%)
Dosing in PJP treatment (N=29)	
Appropriate dose	21 (72.4%)
Underdose	8 (27.6%)
Dosing in *S. maltophilia* treatment (N=14)	
Appropriate dose	1 (7.1%)
Underdose	13 (92.9%)

Regarding the treatment frequency, five (11.6%) received co-trimoxazole four times daily, 25 (58.1%) received it three times daily, 10 (23.3%) received it twice daily, and three (7.0%) received the treatment once daily. All PJP patients received co-trimoxazole at a frequency of administration every six to eight hours (q6-8h), except two patients who received it q12h. The dosing frequency distribution is summarized in Table 3.

**Table 3:** Co-trimoxazole frequency of administration N: number; PJP: *Pneumocystis jirovecii* pneumonia, *S. maltophilia*: *Stenotrophomonas maltophilia*

Frequency	n (%)
Overall (N= 43)	
Four times daily	5 (11.6%)
Three times daily	25 (58.1%)
Twice daily	10 (23.3%)
Once daily	3 (7.0%)
Frequency in PJP treatment (N=29)	
Four times daily	5 (17.2%)
Three times daily	22 (75.9%)
Twice daily	2 (6.9%)
Once daily	0 (0%)
Frequency in *S. maltophilia* treatment (N=14)	
Four times daily	0 (0%)
Three times daily	3 (21.4%)
Twice daily	8 (57.1%)
Once daily	3 (21.4%)

## Discussion

This descriptive study was conducted to assess the appropriateness of co-trimoxazole weight-based dosing in adults with PJP and *S. maltophilia* infections. To the best of our knowledge, this is the first study evaluating the appropriateness of co-trimoxazole dosing in indications requiring weight-based dosing in adults. We found that underdosing of co-trimoxazole occurred at a surprisingly high rate across both the PJP treatment group (27.6%) and the *S. maltophilia* group (92.9%). This could be a result of dosing miscalculation at several points during the prescription procedure. These include determining the optimal dose for a certain indication and calculating the dosage depending on the trimethoprim component.

Co-trimoxazole was likely underdosed in this study for PJP infections because the recommended dosage range for this indication was not used, rather than miscalculating the dose based on the TMP/SMX combination or the TMP component alone. The lowest dose used in our study for PJP infections (in a normal kidney function patient) was 5.5 mg/kg/day of TMP. If the aforementioned confusion occurred, the dose would be close to one-sixth of the recommended dosing range of 15-20 mg/kg/day (i.e., close to 2.5-3.3 mg/kg/day). However, this was not the case for *S. maltophilia* infections. The co-trimoxazole dose was deemed appropriate only in one case, with 92.9% being underdosed. A similar finding was observed in Bakdash and Elajez’s study, which found that 75% of *S. maltophilia* cases were treated with inappropriate weight-based dosing both in adults and pediatrics [13]. More studies are needed to determine whether high doses of co-trimoxazole are truly needed for *S. maltophilia*.

Some pharmacists may also lack knowledge on how to dose co-trimoxazole based on weight. A questionnaire conducted to evaluate the knowledge of community pharmacists about appropriate dosing of antibiotics in pediatrics showed that 84% of pharmacists did not dose co-trimoxazole correctly in pediatrics, which is also dosed based on weight [14]. Another study found that co-trimoxazole was more often underdosed in comparison with other antibiotics in pediatrics [15]. That would suggest co-trimoxazole weight-based dosing, whether in children or in specific indications in adults, may lead to dosing errors. To ensure the appropriateness of weight-based dosing of co-trimoxazole, the dosing of the trimethoprim moiety should be consistent with clinical dosing references and guidelines. An indication of co-trimoxazole should be written on the prescription. If weight-based dosing is warranted, the dosing of TMP should be specified. This would allow the pharmacist to calculate and verify the dose and dispense accordingly. Continuous education on how to calculate combined antibiotics based on weight should be conducted for all healthcare providers, including pharmacists. The majority of PJP patients received the recommended frequency of administration of co-trimoxazole.

Our study has some limitations. Some are due to the retrospective chart review and observational study design. The sample size was relatively small, and we believed this was mainly because co-trimoxazole is not used in adults based on mg/kg dosing except in limited indications, unlike in pediatrics. Also, this study was conducted in a single center, and the prescribing patterns might not be the same as in other hospitals in Saudi Arabia. Despite these limitations, we think the results of this study will be valuable to other institutions, particularly since we could not find other studies published by hospitals in the region. With the rise in antimicrobial resistance, it is the responsibility of every healthcare provider to ensure that antimicrobials are prescribed and administered effectively, reducing the risk of treatment failure and antimicrobial resistance spreading due to improper use or underdosing.

## Conclusions

This study at a Saudi academic hospital was the first to evaluate the appropriateness of co-trimoxazole dosing in indications requiring weight-based dosing in hospitalized adults. The study found a relatively high rate of underdosing of co-trimoxazole in adults with PJP and *S. maltophilia* infections, which are typically treated with higher-than-normal doses. The requirement of weight-based dosing of co-trimoxazole for treating such infections may contribute to the noticed discrepancy. Moreover, dosing the medication based on the trimethoprim moiety alone may enhance the chance of dosing error. More effort should be made to reduce the risk of dosing errors in co-trimoxazole dosing in adults based on weight. Indications of co-trimoxazole need to be provided on the prescription, and dosing based on the trimethoprim moiety should be consistent with clinical dosing references and guidelines. Additionally, education on the calculation of combined antibiotics based on weight is crucial for healthcare providers.
